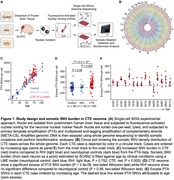# Diverse genomic alterations in single neurons after chronic brain trauma

**DOI:** 10.1002/alz.091891

**Published:** 2025-01-03

**Authors:** Michael B Miller, Chanthia Ma, Guanlan Dong, Samuel Naik, Gannon McDonough, Shulin Mao, Ann C. McKee, August Yue Huang, Eunjung Alice Lee, Christopher A Walsh

**Affiliations:** ^1^ Brigham and Women’s Hospital, Boston, MA USA; ^2^ Harvard Medical School, Boston, MA USA; ^3^ Boston University Chobanian & Avedisian School of Medicine, Boston, MA USA; ^4^ Boston Children’s Hospital, Boston, MA USA

## Abstract

**Background:**

Chronic traumatic encephalopathy (CTE) is a neurodegenerative disease associated with repetitive head impact (RHI) although little is known about its molecular pathogenesis. Previous studies of single neurons showed that private somatic mutations increase both during normal aging and in neurodegenerative disorders, and show diverse mutational patterns.

**Method:**

We applied two orthogonal single‐nucleus whole‐genome sequencing (snWGS) methods to neurons isolated from the prefrontal cortex of 15 individuals with CTE, and 4 individuals with RHI but no CTE diagnosis, and compared mutational rates and spectra with neurons from neurotypical controls and Alzheimer’s disease (AD).

**Result:**

We found a modest but significant elevation of somatic double‐stranded single‐nucleotide variants (SNVs) that resembles a pattern previously reported in AD. In addition, we found a strikingly large burden of small insertions and deletions (indels) and used duplex sequencing to show that these indels are mainly single‐stranded, and again found a similar phenomenon in neurons from AD brain.

**Conclusion:**

Our results suggest that neurons in CTE brain are exposed to stereotyped mutational processes, and that these processes are shared between AD and CTE suggesting potentially shared pathogenic mechanisms. Furthermore, the absence of similar changes in RHI neurons without CTE suggests that the development of CTE entails a mechanism beyond that caused by RHI alone.